# *rac*-1-Acetyl-5-benzyl-2-thioxoimidazolidin-4-one

**DOI:** 10.1107/S1600536808041883

**Published:** 2008-12-13

**Authors:** Mary C. Uzcátegui, Gerzon E. Delgado, Asiloé J. Mora, Teresa González, Alexander Briceño

**Affiliations:** a Laboratorio de Cristalografía, Departamento de Química, Facultad de Ciencias, Universidad de Los Andes, Mérida 5101, Venezuela; b Centro de Química, Instituto Venezolano de Investigaciones, Científicas (IVIC), Apartado 21827, Caracas 1020-A, Venezuela

## Abstract

In the title compound, C_12_H_12_N_2_O_2_S, the mol­ecules have a wing-like conformation, with a distance of 3.797 (2) Å between the centroids of the five- and six-membered rings. In the crystal structure, mol­ecules are linked by N—H⋯O hydrogen bonds, forming infinite one-dimensional zigzag chains, running along [001], with a *C*(4) graph-set motif.

## Related literature

For related compounds, see: Seijas *et al.* (2006 ▶, 2007 ▶); Delgado *et al.* (2007 ▶); Sulbaran *et al.* (2007 ▶). For racemization of amino acids, see: Yamada *et al.* (1983 ▶); Yoshioka (2007 ▶). For reference structural data, see: Allen *et al.* (2002 ▶). For hydrogen-bond motifs in graph-set notation, see Etter (1990 ▶).
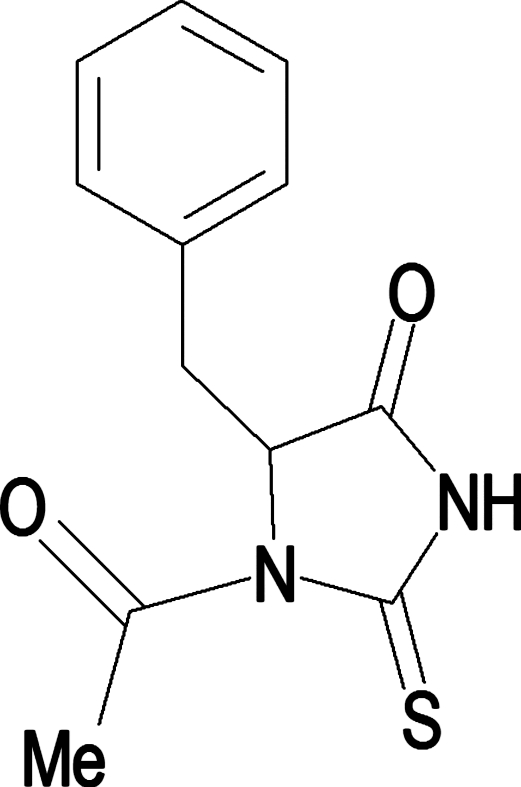


## Experimental

### 

#### Crystal data

C_12_H_12_N_2_O_2_S
*M* *_r_* = 248.30
Monoclinic, 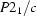

*a* = 11.696 (5) Å
*b* = 13.479 (6) Å
*c* = 7.767 (4) Å
β = 94.41 (1)°
*V* = 1220.8 (9) Å^3^
*Z* = 4
Mo *K*α radiation
μ = 0.26 mm^−1^
*T* = 298 (2) K
0.4 × 0.3 × 0.2 mm

#### Data collection

Rigaku AFC-7S Mercury diffractometer
Absorption correction: multi-scan (Jacobson, 1998 ▶) *T*_min_ = 0.900, *T*_max_ = 0.950
12945 measured reflections
2349 independent reflections
2065 reflections with *I* > 2σ(*I*)
*R*_int_ = 0.026

#### Refinement

*R*[*F* ^2^ > 2σ(*F* ^2^)] = 0.045
*wR*(*F* ^2^) = 0.124
*S* = 1.05
2349 reflections
156 parameters
H-atom parameters constrained
Δρ_max_ = 0.24 e Å^−3^
Δρ_min_ = −0.27 e Å^−3^

### 

Data collection: *CrystalClear* (Rigaku, 2002 ▶); cell refinement: *CrystalClear*; data reduction: *CrystalStructure* (Rigaku/MSC, 2004 ▶); program(s) used to solve structure: *SHELXS97* (Sheldrick, 2008 ▶); program(s) used to refine structure: *SHELXL97* (Sheldrick, 2008 ▶); molecular graphics: *DIAMOND* (Brandenburg, 1999 ▶); software used to prepare material for publication: *PLATON* (Spek, 2003 ▶) and *publCIF* (Westrip, 2009 ▶).

## Supplementary Material

Crystal structure: contains datablocks I, global. DOI: 10.1107/S1600536808041883/cv2495sup1.cif

Structure factors: contains datablocks I. DOI: 10.1107/S1600536808041883/cv2495Isup2.hkl

Additional supplementary materials:  crystallographic information; 3D view; checkCIF report

## Figures and Tables

**Table 1:** Hydrogen-bond geometry (Å, °)

*D*—H⋯*A*	*D*—H	H⋯*A*	*D*⋯*A*	*D*—H⋯*A*
N3—H3⋯O4^i^	0.86	1.98	2.834 (2)	175

Symmetry code: (i) 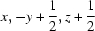.

**Fig. 1.**
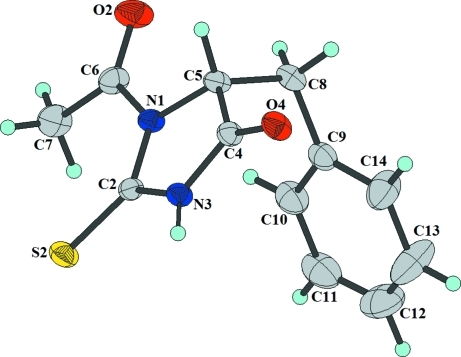
The molecular structure of (I), showing the atomic numbering scheme. Displacement elipsoids are drawn at the 25% probability level and H atoms are shown as spheres of arbitrary radii.

**Fig. 2.**
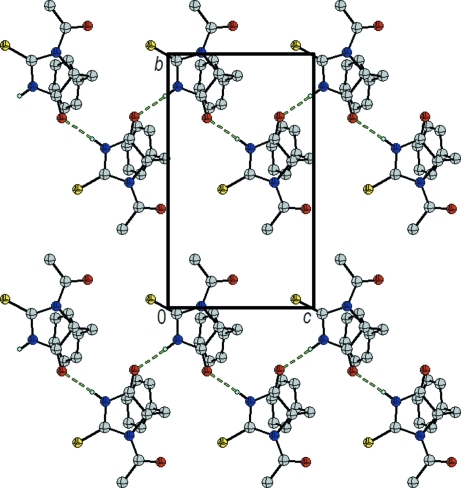
A portion of the crystal packing viewed along the a-axis. Hydrogen bonds are marked with dashed lines. H atoms not involved in hydrogen bonding have been omitted for clarity.

## supplementary crystallographic information

### Comment

In continuation of our study of N-carbamoyl, hydantoin and thiohydantoin derivatives of α-amino acids (Seijas *et al.*, 2006, 2007; Delgado *et al.*, 2007; Sulbaran *et al.*, 2007), we report here the structure of the title compound (I) - the *N*-acetylthiohydantoin derivative of the α-amino acid *L*-phenylalanine.

Compound (I) (Fig. 1) crystallizes in a centrosymmetric space group, which implies that *L*-phenylalanine suffered an amino acid racemization produced by the use of acetic acid in the synthesis (Yamada *et al.* 1983; Yoshioka, 2007). All bond distances and angles are normal (Allen, 2002). The thiohydantoin ring is essentially planar with a maximum deviations of 0.023 (1) Å in C4 and -0.025 (2) Å in C5. The molecular structure and crystal packing of (I) are stabilized by intermolecular N3—H3···O4 (*x*, 1/2 - *y*, 1/2 + *z*) hydrogen bonds (Table 1), forming infinite one-dimentional zigzag chains that run along [001] direction, which can be described in graph-set notation as C(4) (Etter, 1990) (Figure 2).

### Experimental

*L*-phenylalanine (3.4 mmol) and NH_4_SCN (3.4 mmol) was dissolved in a 9 ml acetic anhydride - 1 ml acetic acid mixture and transferred in a round-bottom flask. The mixture was warmed, with agitation, to 363 K over a period of 30 min. The resulting solution was cooled in a ice/water mixture and stored in a freezer overnight. The resulting white solid was filtered off and washed with cool water (m.p. 441–443 K). Crystal of (I) suitable for X-ray diffraction analysis were obtained by slow evaporation of a 1:1 ethanol-methanol solution.

### Refinement

All H atoms were placed at calculated positions and treated using the riding model, with C—H distances of 0.93–0.98 A, and N—H distances of 0.86 A. The *U*_iso_(H) parameters were fixed at 1.2*U*_eq_(C, N) and 1.5*U*_eq_(methyl).

### Crystal data

**Table:**

C_12_H_12_N_2_O_2_S	*F*(000) = 520
*M**_r_* = 248.30	*D*_x_ = 1.351 Mg m^−^^3^
Monoclinic, *P*2_1_/*c*	Melting point = 441–443 K
Hall symbol: -P 2ybc	Mo *K*α radiation, λ = 0.71070 Å
*a* = 11.696 (5) Å	Cell parameters from 4020 reflections
*b* = 13.479 (6) Å	θ = 2.4–27.8°
*c* = 7.767 (4) Å	µ = 0.26 mm^−^^1^
β = 94.41 (1)°	*T* = 298 K
*V* = 1220.8 (9) Å^3^	Block, colourless
*Z* = 4	0.4 × 0.3 × 0.2 mm

### Data collection

**Table:**

Rigaku AFC-7S Mercury diffractometer	2349 independent reflections
Radiation source: Normal-focus sealed tube	2065 reflections with *I* > 2σ(*I*)
graphite	*R*_int_ = 0.026
Detector resolution: 14.6306 pixels mm^-1^	θ_max_ = 28.0°, θ_min_ = 2.3°
ω scans	*h* = −13→13
Absorption correction: multi-scan (Jacobson, 1998)	*k* = −15→15
*T*_min_ = 0.900, *T*_max_ = 0.950	*l* = −9→6
12945 measured reflections	

### Refinement

**Table:**

Refinement on *F*^2^	Secondary atom site location: difference Fourier map
Least-squares matrix: full	Hydrogen site location: inferred from neighbouring sites
*R*[*F*^2^ > 2σ(*F*^2^)] = 0.045	H-atom parameters constrained
*wR*(*F*^2^) = 0.124	*w* = 1/[σ^2^(*F*_o_^2^) + (0.0616*P*)^2^ + 0.4929*P*] where *P* = (*F*_o_^2^ + 2*F*_c_^2^)/3
*S* = 1.05	(Δ/σ)_max_ = 0.001
2349 reflections	Δρ_max_ = 0.24 e Å^−^^3^
156 parameters	Δρ_min_ = −0.27 e Å^−^^3^
0 restraints	Extinction correction: *SHELXL97* (Sheldrick, 2008), Fc^*^=kFc[1+0.001xFc^2^λ^3^/sin(2θ)]^-1/4^
Primary atom site location: structure-invariant direct methods	Extinction coefficient: 0.013 (2)

### Special details

**Table:**

Geometry. All e.s.d.'s (except the e.s.d. in the dihedral angle between two l.s. planes) are estimated using the full covariance matrix. The cell e.s.d.'s are taken into account individually in the estimation of e.s.d.'s in distances, angles and torsion angles; correlations between e.s.d.'s in cell parameters are only used when they are defined by crystal symmetry. An approximate (isotropic) treatment of cell e.s.d.'s is used for estimating e.s.d.'s involving l.s. planes.
Refinement. Refinement of *F*^2^ against ALL reflections. The weighted *R*-factor *wR* and goodness of fit *S* are based on *F*^2^, conventional *R*-factors *R* are based on *F*, with *F* set to zero for negative *F*^2^. The threshold expression of *F*^2^ > σ(*F*^2^) is used only for calculating *R*-factors(gt) *etc*. and is not relevant to the choice of reflections for refinement. *R*-factors based on *F*^2^ are statistically about twice as large as those based on *F*, and *R*- factors based on ALL data will be even larger.

### Fractional atomic coordinates and isotropic or equivalent isotropic displacement parameters (Å^2^)

**Table:**

	*x*	*y*	*z*	*U*_iso_*/*U*_eq_	
S2	0.84958 (5)	0.53330 (4)	0.61947 (7)	0.0501 (2)	
O2	0.88296 (18)	0.61004 (12)	0.0516 (2)	0.0689 (5)	
O4	0.87718 (14)	0.24785 (10)	0.23095 (18)	0.0512 (4)	
N1	0.85248 (14)	0.50588 (12)	0.26762 (19)	0.0366 (4)	
N3	0.86134 (14)	0.37142 (11)	0.42952 (19)	0.0383 (4)	
H3	0.8622	0.3343	0.5196	0.046*	
C2	0.85359 (16)	0.47307 (13)	0.4365 (2)	0.0356 (4)	
C4	0.86755 (17)	0.33477 (14)	0.2669 (2)	0.0378 (4)	
C5	0.85594 (17)	0.42208 (14)	0.1459 (2)	0.0385 (4)	
H5	0.9237	0.4272	0.0796	0.046*	
C6	0.86200 (19)	0.60326 (15)	0.2013 (3)	0.0476 (5)	
C7	0.8434 (2)	0.69011 (16)	0.3128 (3)	0.0626 (7)	
H7A	0.8421	0.7495	0.2445	0.094*	
H7B	0.9046	0.6941	0.4024	0.094*	
H7C	0.7716	0.6829	0.3637	0.094*	
C8	0.74690 (19)	0.41292 (17)	0.0231 (3)	0.0487 (5)	
H8A	0.7375	0.4732	−0.0446	0.058*	
H8B	0.7565	0.3585	−0.0561	0.058*	
C9	0.63988 (19)	0.39550 (17)	0.1147 (3)	0.0496 (5)	
C10	0.5823 (2)	0.4733 (2)	0.1867 (3)	0.0634 (7)	
H10	0.6101	0.5376	0.1786	0.076*	
C11	0.4837 (3)	0.4563 (3)	0.2707 (4)	0.0837 (10)	
H11	0.4463	0.5092	0.3190	0.100*	
C12	0.4967 (3)	0.2862 (3)	0.2141 (7)	0.1180 (15)	
H12	0.4681	0.2223	0.2237	0.142*	
C13	0.4415 (3)	0.3629 (4)	0.2826 (5)	0.1022 (12)	
H13	0.3749	0.3518	0.3378	0.123*	
C14	0.5954 (3)	0.3015 (2)	0.1296 (5)	0.0825 (9)	
H14	0.6319	0.2478	0.0826	0.099*	

### Atomic displacement parameters (Å^2^)

**Table:**

	*U*^11^	*U*^22^	*U*^33^	*U*^12^	*U*^13^	*U*^23^
S2	0.0680 (4)	0.0440 (4)	0.0399 (3)	−0.0022 (2)	0.0150 (2)	−0.0088 (2)
O2	0.1084 (15)	0.0490 (10)	0.0522 (10)	0.0001 (9)	0.0243 (9)	0.0161 (7)
O4	0.0777 (11)	0.0330 (8)	0.0435 (8)	0.0035 (7)	0.0093 (7)	−0.0038 (6)
N1	0.0462 (10)	0.0304 (8)	0.0341 (8)	0.0014 (7)	0.0091 (6)	0.0021 (6)
N3	0.0531 (10)	0.0312 (8)	0.0312 (8)	−0.0013 (7)	0.0075 (6)	0.0022 (6)
C2	0.0369 (10)	0.0348 (10)	0.0358 (10)	−0.0015 (7)	0.0081 (7)	0.0009 (7)
C4	0.0439 (11)	0.0342 (10)	0.0356 (10)	0.0009 (8)	0.0067 (7)	−0.0014 (7)
C5	0.0491 (12)	0.0344 (10)	0.0335 (10)	0.0021 (8)	0.0118 (8)	0.0006 (7)
C6	0.0571 (14)	0.0352 (11)	0.0516 (13)	0.0004 (9)	0.0110 (10)	0.0083 (9)
C7	0.0881 (19)	0.0330 (12)	0.0679 (16)	0.0020 (11)	0.0140 (13)	0.0055 (10)
C8	0.0588 (14)	0.0552 (13)	0.0319 (10)	0.0042 (10)	0.0029 (9)	−0.0021 (9)
C9	0.0471 (13)	0.0623 (14)	0.0385 (11)	0.0045 (10)	−0.0029 (8)	−0.0030 (9)
C10	0.0547 (15)	0.0767 (19)	0.0582 (15)	0.0128 (12)	−0.0001 (11)	−0.0111 (12)
C11	0.0587 (18)	0.126 (3)	0.0657 (18)	0.0246 (18)	0.0017 (13)	−0.0179 (18)
C12	0.070 (2)	0.102 (3)	0.186 (4)	−0.022 (2)	0.034 (3)	0.016 (3)
C13	0.060 (2)	0.144 (4)	0.106 (3)	0.000 (2)	0.0263 (18)	0.009 (2)
C14	0.0598 (17)	0.0713 (19)	0.118 (3)	−0.0079 (14)	0.0165 (16)	−0.0138 (17)

### Geometric parameters (Å, °)

**Table:**

S2—C2	1.6402 (19)	C7—H7C	0.9600
O2—C6	1.210 (3)	C8—C9	1.505 (3)
O4—C4	1.212 (2)	C8—H8A	0.9700
N1—C2	1.384 (2)	C8—H8B	0.9700
N1—C6	1.418 (2)	C9—C14	1.378 (4)
N1—C5	1.476 (2)	C9—C10	1.387 (3)
N3—C4	1.363 (2)	C10—C11	1.387 (4)
N3—C2	1.374 (2)	C10—H10	0.9300
N3—H3	0.8600	C11—C13	1.358 (5)
C4—C5	1.506 (3)	C11—H11	0.9300
C5—C8	1.537 (3)	C12—C13	1.349 (5)
C5—H5	0.9800	C12—C14	1.386 (5)
C6—C7	1.482 (3)	C12—H12	0.9300
C7—H7A	0.9600	C13—H13	0.9300
C7—H7B	0.9600	C14—H14	0.9300
			
C2—N1—C6	130.19 (17)	H7B—C7—H7C	109.5
C2—N1—C5	111.36 (15)	C9—C8—C5	113.59 (16)
C6—N1—C5	117.97 (16)	C9—C8—H8A	108.8
C4—N3—C2	113.97 (15)	C5—C8—H8A	108.8
C4—N3—H3	123.0	C9—C8—H8B	108.8
C2—N3—H3	123.0	C5—C8—H8B	108.8
N3—C2—N1	106.08 (15)	H8A—C8—H8B	107.7
N3—C2—S2	122.29 (14)	C14—C9—C10	117.5 (2)
N1—C2—S2	131.63 (15)	C14—C9—C8	121.1 (2)
O4—C4—N3	125.20 (18)	C10—C9—C8	121.3 (2)
O4—C4—C5	128.11 (17)	C9—C10—C11	120.9 (3)
N3—C4—C5	106.65 (16)	C9—C10—H10	119.6
N1—C5—C4	101.76 (14)	C11—C10—H10	119.6
N1—C5—C8	113.36 (16)	C13—C11—C10	120.3 (3)
C4—C5—C8	110.80 (17)	C13—C11—H11	119.9
N1—C5—H5	110.2	C10—C11—H11	119.9
C4—C5—H5	110.2	C13—C12—C14	120.9 (4)
C8—C5—H5	110.2	C13—C12—H12	119.5
O2—C6—N1	116.53 (19)	C14—C12—H12	119.5
O2—C6—C7	123.47 (19)	C12—C13—C11	119.8 (3)
N1—C6—C7	119.98 (18)	C12—C13—H13	120.1
C6—C7—H7A	109.5	C11—C13—H13	120.1
C6—C7—H7B	109.5	C12—C14—C9	120.7 (3)
H7A—C7—H7B	109.5	C12—C14—H14	119.7
C6—C7—H7C	109.5	C9—C14—H14	119.7
H7A—C7—H7C	109.5		

### Hydrogen-bond geometry (Å, °)

**Table:**

*D*—H···*A*	*D*—H	H···*A*	*D*···*A*	*D*—H···*A*
N3—H3···O4^i^	0.86	1.98	2.834 (2)	175

Symmetry codes: (i) *x*, −*y*+1/2, *z*+1/2.
